# Attitude of healthcare professionals in Pakistan towards inter-professional collaborative competencies: A cross-sectional study

**DOI:** 10.1097/MD.0000000000049107

**Published:** 2026-07-17

**Authors:** Seemab Ashraf, Azhar Hussain, Madeeha Malik, Ayman M. Al-Qaaneh, Ayisha Hashmi, Suresh Shanmugham, Shazia Jamshed, Long Chiau Ming, Muhammad Mamoon Iqbal, Ayesha Iqbal, Mohamed N. Al-Arifi, Wajid Syed, Mahmood Basil A. Alrawi

**Affiliations:** a Hamdard Institute of Pharmaceutical Sciences, Hamdard University Islamabad, Islamabad, Pakistan; b Pak-Austria Fachhochschule: Institute of Applied Sciences and Technology, Haripur, Pakistan; c Cyntax Health Projects, Contract Research Organization, Islamabad, Pakistan; d Department of Allied Health Sciences, Al-Balqa Applied University (BAU), City of Al-Salt, Jordan; e Department of Pharmacy Practice, School of Pharmacy, IMU (Former International Medical University), Kuala Lumpur, Malaysia; f Department of Pharmacy Practice, Faculty of Pharmacy, Jinnah University for Women, Karachi, Pakistan; g Department of Pharmacy Practice, Shifa College of Pharmaceutical Sciences, Shifa Tameer-e-Millat University, Islamabad, Pakistan; h Department of Biomedical Sciences, Sir Jeffrey Cheah Sunway Medical School, Faculty of Medical and Life Sciences, Sunway University, Kuala Lumpur, Malaysia; i Department of Respiratory Sciences, Institute for Lung Health, University of Leicester, Leicester, UK; j Department of Respiratory Medicine, National Institute for Health Research Leicester Biomedical research Centre- Respiratory, University Hospitals of Leicester NHS Trust, Glenfield Hospital, Leicester, UK; k Department of Pharmacy Practice and Policy, University Park Campus, University of Nottingham, Nottingham, United Kingdom; l Office of Lifelong Learning and the Physician Learning Program, Faculty of Medicine and Dentistry, University of Alberta, AB T6G1C9, Edmonton, Canada; m Department of Clinical Pharmacy, college of pharmacy, King Saud University, Riyadh, Saudi Arabia; n Department of Optometry, College of applied medical sciences King Saud University, Riyadh, Saudi Arabia.

**Keywords:** healthcare professionals, inter-professional education (IPE), inter-professional education collaborative (IPEC) competencies, nurses, Pakistan, pharmacists, physicians

## Abstract

Inter-professional education (IPE) is recognized as best-suited strategy to improve health professionals’ collaborative competencies, leading to improved patient outcomes. However, assessment of attitude of healthcare professionals towards these competencies is also important for developing effective strategies for promoting inter-professional collaboration. The present study was designed to assess IPE collaborative (IPEC) competencies of healthcare professionals including physicians, nurses and pharmacists in Pakistan. A descriptive cross-sectional study design was used to assess IPEC competencies of 500 healthcare professionals i.e. (physicians, nurses, and pharmacists) working in tertiary care healthcare facilities located in 4 cities of Pakistan were selected using convenient sampling technique. A pre-validated tool IPEC self-assessment was used for data collection. After data collection, data was analyzed statistically. The mean scores of healthcare professionals regarding IPEC Competencies were calculated for total items in which mean score of nurses was highest (67.34 ± 0.52), followed by pharmacists (66.24 ± 0.58) and then doctors (65.88 ± 0.44). The results depicted higher IPEC competencies in all the health professionals. On both the domains i.e., inter-professional values and inter-professional interaction, nurses outscored both pharmacists and doctors with mean scores 34.46 ± 0.26 and 32.88 ± 0.28 respectively. The findings indicate that healthcare professionals in Pakistan generally demonstrate positive attitudes toward inter-professional collaborative competencies, with nurses showing comparatively higher scores across both inter-professional values and inter-professional interaction domains. Although the overall competency levels were encouraging, the variation across professional groups suggests the need for more targeted efforts to strengthen collaborative practice among all disciplines. Integrating structured IPE and training into clinical and academic settings may help bridge these gaps and further enhance teamwork, ultimately supporting better patient care outcomes.

## 1. Introduction

World Health Organization^[1]^ has emphasized on the need of collaborative patient care and has recognized inter-professional education (IPE) as the best-suited strategy to improve health professionals’ collaborative competencies, thus, leading to quality patient outcomes. Keeping in view the recommended solution, IPE collaborative (IPEC), the expert panel in the United States has designed 4 core collaborative competencies which can serve as a guide to prepare healthcare professionals for practicing IPC. These are required to improve teamwork and cooperation among health professionals from different disciplines with patients, their families, and the community overall. These core competencies include ethics or values, roles or responsibilities, inter-professional communication, and teams and teamwork. These competencies should be assessed in healthcare professionals so that further measures can be taken to develop and foster inter-professional collaboration (IPC) in the healthcare system of the country.^[2–4]^

Interprofessional communication is consistently identified as a central component of collaborative practice. A study conducted in the United States highlighted communication as the most essential IPEC competency.^[5]^ Similarly, E Suter, J Arndt, N Arthur, J Parboosingh, E Taylor and S Deutschlander^[6]^ emphasized that effective communication, alongside a clear understanding of professional roles, forms the foundation of IPC. Evidence further suggests that strong communication practices are directly associated with improved provider performance and better patient outcomes. However, findings from New Zealand indicate that while healthcare professionals generally agree on the relevance of these competencies, gaps persist in their practical implementation and educational integration.^[7]^

With regard to roles and responsibilities, clarity in professional functions remains a critical factor in achieving effective collaboration. Research from Germany demonstrated that both physicians and nurses recognize the importance of IPC in clinical settings. However, the pathway through which these competencies are developed differs between professions. Physicians often acquire collaborative skills through clinical experience, whereas nurses tend to develop them during their formal education.^[8]^ This variation highlights the need for more consistent and structured inclusion of IPE within academic curricula.

The domains of values and ethics, as well as teamwork, also play a key role in shaping collaborative practice. A qualitative study conducted in Singapore found that inter-professional interactions are strongly influenced by workplace culture, mutual respect, and openness to shared learning.^[9,10]^ Overcoming hierarchical barriers and fostering respect among professionals were identified as essential for improving collaboration. In addition, teamwork has been shown to be fundamental for ensuring patient safety, accountability, and quality of care. Evidence from emergency care settings indicates that experience within a team environment significantly influences collaborative attitudes, even when demographic factors such as age or gender do not (Plummer & Copnell, 2016). Notably, physicians in some settings were found to hold less favorable views toward collaboration, reinforcing the need for targeted educational interventions.^[9]^

From a broader perspective, the impact of IPC on patient outcomes has been well documented. A recent meta-analysis from Ethiopia reported that collaboration between nurses and physicians remains at a moderate level and that poor collaboration can lead to serious consequences, including increased medical errors, hospital-acquired infections, and higher healthcare costs.^[11]^ The study recommended practical strategies such as training programs, improved communication systems, and team-building initiatives to strengthen collaborative practice. Despite the growing body of international evidence, most research on IPEC competencies has been conducted in developed countries. There remains a noticeable lack of data from developing contexts, including Pakistan. Given the differences in healthcare systems, educational structures, and workplace dynamics, it is important to assess how healthcare professionals in such settings perceive inter-professional collaborative competencies. Addressing this gap can support the development of context-specific strategies to enhance collaboration and ultimately improve patient care outcomes. Therefore, the present study was designed to assess IPEC Competencies of healthcare professionals including physicians, nurses and pharmacists in Pakistan.

## 2. Methodology

### 2.1. Study design

A descriptive cross-sectional study design was used to assess IPEC Competencies of healthcare professionals including physicians, nurses, and pharmacists in Pakistan. For data collection, permission was taken from the Medical Superintendent of different public and private hospitals. Participation in the study was voluntary. Written informed consent was received from the participants assuring anonymity and confidentiality of their responses. Data was collected during November 2022 to April 2023. The inclusion criteria were healthcare professionals (physicians, nurses, and pharmacists) working in tertiary care hospitals, with at least 1 year of clinical experience, and willing to participate in the study. The HCPs were drawn from multiple clinical settings, including Medical, Surgical, Gynecology, Pharmacy, Orthopedic, Oncology, Psychiatry, and Cardiology. The exclusion criteria included healthcare professionals not directly involved in patient care, interns or trainees without independent clinical responsibilities, and those who declined participation or provided incomplete response.

### 2.2. Sample size and sampling technique

This study was conducted in 20 different hospitals in Pakistan. Healthcare professionals selected for this study were physicians, nurses, and pharmacists working in different hospitals located in Lahore, Islamabad, Rawalpindi, and Taxila. Participants were recruited from different departments of the hospitals mainly medical, surgical, gynecology, and pharmacy. According to World Health Organization sample size criteria, twenty healthcare facilities were randomly selected from the list of tertiary care healthcare facilities obtained from the District Health Office. Thirty healthcare professionals were selected from each facility using convenient sampling technique making a total sample of 600 healthcare professionals i.e. physicians (n = 200), nurses (n = 200), and pharmacists (n = 200). Out of the 600 healthcare professionals invited, 500 completed the questionnaire, resulting in a response rate of 83.3%. These participants formed the final analytical sample, comprising physicians (n = 200), nurses (n = 200), and pharmacists (n = 100), while the overall nonresponse rate was 16.7%. The lower participation among pharmacists was mainly due to their limited availability across the selected hospitals, as pharmacy staffing levels varied between institutions. In addition, some pharmacists were unable to participate because of workload constraints and time limitations during the data collection period.

### 2.3. Study tool

To measure IPEC competencies of health professionals, a revised 16-item IPEC self-assessment tool was used^[12]^ (Cronbach alpha = 0.90). It a 5-point Likert scale ranging from strongly disagree to strongly agree (1–5). This tool comprises of 2 main domains i.e., inter-professional interaction and inter-professional values. The domain of Interprofessional Interactions focuses on the practical aspects of teamwork in clinical settings. This includes the ability to communicate effectively with other health professionals, engage in shared problem-solving, integrate input from different disciplines into patient care decisions, manage disagreements constructively, apply leadership in team settings, and use strategies that strengthen teamwork and collaboration. It also reflects skills related to using evidence in team-based care and developing trust among team members. Interprofessional Values focuses on the attitudes and professional principles that support collaborative practice.^[12]^ This includes placing patients at the center of care, respecting confidentiality, valuing diversity within healthcare teams, demonstrating ethical conduct, maintaining honesty and integrity in professional relationships, understanding the roles and responsibilities of other health professions, and respecting the cultures and values of different disciplines.^[12]^ It also emphasizes maintaining one’s own professional competence. Higher scores on the scale represent higher collaborative competencies of the healthcare professional. The minimum score of the scale is 16 while the maximum score is 80.^[3]^ Pilot testing was conducted to check the reliability of the tool for which Cronbach alpha value was found to be 0.752.

Data was collected from healthcare professionals (physicians, nurses, pharmacists) working in different hospitals in Pakistan. Participants were reached at the hospitals and were briefed about the research study by the principal researcher. Questionnaires were self-administered by the participants and were collected from the respondents after completion.

### 2.4. Data analysis

After data collection and manual data entry was performed and the responses of the HCPs were analyzed using IBM statistical package for social science version 27. Prior to analysis normality of the data was assessed using the Shapiro–Wilk test, which indicated that the data were not normally distributed (*P* <.05). Based on this finding, nonparametric statistical tests were used. Accordingly, the Mann–Whitney *U* test was used for comparisons between 2 independent groups, while the Kruskal–Wallis test was applied for comparisons involving more than 2 independent groups. At a statistical significance of *P* ≤.05 were used.

## 3. Results

### 3.1. Demographic characteristics of healthcare professionals

Out of 500 healthcare professionals, 27.8% (n = 139) were males and 72.2% (n = 361) were females. Regarding age, 30.6% (n = 153) were in the age group 21 to 25 years, 40.2% (n = 201) were of 26 to 30 years. Seventy 5 percent of health care professionals were working in the hospitals of Lahore. Out of 500 health professionals, 40% were doctors (n = 200), 40% were nurses (n = 200) and 20% were pharmacists (n = 100). Around three fifth (n = 305, 61%) of the health care professionals had experience of up to 5 years (n = 305). Regarding the wards of the hospitals, half of the respondents were from medical (n = 146, 29.2%) and surgical (n = 122, 24.4%) wards. A detailed description is given (Table 1).

**Table 1:** Demographic characteristics of healthcare professionals.

Variable		n (%)
Gender	Male	139 (27.8)
	Female	361 (72.2)
Age in years	21–25	153 (30.6)
	26–30	201 (40.2)
	31–35	77 (15.4)
	36–40	43 (8.6)
	41–45	13 (2.6)
	46–50	5 (1.0)
	Above 50	8 (1.6)
Qualification	Graduate	378 (75.6)
	Masters/M.Phil./FCPS	119 (23.8)
	Doctorate	3 (.6)
Hospital location	Lahore	375 (75.0)
	Islamabad	78 (15.6)
	Rawalpindi	25 (5.0)
	Taxila	22 (4.4)
Profession	Doctor	200 (40.0)
	Pharmacist	100 (20.0)
	Nurse	200 (40.0)
Years of experience	1–5 yr	305 (61.0)
	6–10 yr	114 (22.8)
	11-15 yr	51 (10.2)
	16–20 yr	17 (3.4)
	More than 20 yr	13 (2.6)
Department/ward	Medical	146 (29.2)
	Surgical	122 (24.4)
	Gynecology	98 (19.6)
	Pharmacy	82 (16.4)
	Orthopedic	3 (.6)
	Oncology	22 (4.4)
	Psychiatry	7 (1.4)
	Cardiology	20 (4.0)

n = frequency; % = percentage.

### 3.2. Assessment of IPEC competencies of healthcare professionals in Pakistan

The results regarding IPEC Competencies of healthcare professionals on inter-professional interaction domain highlighted that 70% (n = 350) of healthcare professionals agreed that they are able to select communication techniques and tools facilitating team interactions. Only 4.2% (n = 21) of respondents disagreed on being able to engage other medical professionals in jointly solving the problems relevant to the situation. More than eighty percent of health professionals agreed that they are able to inform care-related decisions by utilizing the knowledge and experience of other professionals relevant to the situation. Three fifth of the respondents agreed (n = 302, 60.4%) on being able to apply leadership practices supporting effective collaboration. Around sixty percent of respondents (n = 287, 57.4%) agreed that they are able to use strategies for improving IPC and teamwork’s effectiveness as shown in Table 2. More than sixty percent of healthcare professionals (n = 315, 63.0%) showed agreement that they are able to use evidence available in the facility to integrate team-based practices and collaboration. Only 1.2% of professionals (n = 6) disagreed on understanding the expertise, skills, and responsibilities of other health-related professions (Table 2).

**Table 2:** Assessment of IPEC competencies of healthcare professionals in Pakistan.

Variable	Strongly disagree n (%)	Disagree n (%)	Neither agree nor disagree n (%)	Agree n (%)	Strongly agree n (%)
Inter-professional interaction					
I am able to choose communication tools and techniques that facilitate effective team interactions	4 (0.8)	12 (2.4)	52 (10.4)	350 (70.0)	82 (16.4)
I am able to engage other health professionals in shared problem-solving appropriate to the specific care situation	0 (0.0)	21 (4.2)	71 (14.2)	288 (57.6)	120 (24.0)
I am able to inform care decisions by integrating the knowledge and experience of other professions appropriate to the clinical situation	1 (0.2)	14 (2.8)	66 (13.2)	289 (57.8)	130 (26.0)
I am able to apply leadership practices that support effective collaborative practice	2 (0.4)	19 (3.8)	71 (14.2)	302 (60.4)	106 (21.2)
I am able to engage other health professionals to constructively manage disagreements about patient care	2 (0.4)	16 (3.2)	54 (10.8)	315 (63.0)	113 (22.6)
I am able to use strategies that improve the effectiveness of inter-professional teamwork and team-based care	1 (0.2)	9 (1.8)	66 (13.2)	287 (57.4)	137 (27.4)
I am able to use available evidence to inform effective teamwork and team-based practices	4 (0.8)	9 (1.8)	40 (8.0)	315 (63.0)	132 (26.4)
I am able to understand the responsibilities and expertise of other health professions	1 (0.2)	5 (1.0)	22 (4.4)	298 (59.6)	174 (34.8)
Inter-professional values					
I am able to place the interests of patients at the center of inter-professional healthcare delivery	2 (0.4)	6 (1.2)	44 (8.8)	322 (64.4)	126 (25.2)
I am able to respect the privacy of patients while maintaining confidentiality in the delivery of team-based care	1 (0.2)	4 (0.8)	31 (6.2)	261 (52.2)	203 (40.6)
I am able to embrace the diversity that characterizes the healthcare team	0 (0.0)	12 (2.4)	63 (12.6)	328 (65.6)	97 (19.4)
I am able to respect the cultures and values of other health professions	0 (0.0)	4 (0.8)	28 (5.6)	259 (51.8)	209 (41.8)
I am able to develop a trusting relationship with other team members	1 (0.2)	6 (1.2)	26 (5.2)	299 (59.8)	168 (33.6)
I am able to demonstrate high standards of ethical conduct in my contributions to team-based care	2 (0.4)	7 (1.4)	33 (6.6)	286 (57.2)	172 (34.4)
I am able to act with honesty and integrity in relationships with other team members.	5 (1.0)	3 (0.6)	19 (3.8)	283 (56.6)	190 (38.0)
I am able to maintain competence in my own profession appropriate to my level of training.	0 (0.0)	5 (1.0)	16 (3.2)	288 (57.6)	191 (38.2)

IPEC = inter-professional education collaborative.

The results regarding IPEC Competencies of healthcare professionals on inter-professional values domain highlighted that one-fourth of the respondents (n = 126, 25.2%) strongly agreed on being able to place patients’ interests as foremost in the inter-professional delivery of healthcare. More than ninety percent of health professionals agreed on their ability to respect patients’ privacy along with the maintenance of confidentiality in delivering collaborative patient care. More than half of the respondents (n = 259, 51.8%) agreed that they are able to respect the values and cultures of other healthcare professions. Only 1.6% (n = 8) of respondents reported disagreement on being able to act with integrity and honesty with other members in the team. More than one-third of health professionals (n = 191, 38.2%) strongly agreed on being able to stay competent in their professional practice appropriate to their training level. A detailed description is given (Table 2).

### 3.3. Mean scores of healthcare professionals regarding IPEC competencies

The mean scores of healthcare professionals regarding IPEC Competencies were calculated for total items the mean scores of healthcare professionals regarding IPEC competencies were calculated for total items. The mean score of nurses was highest (67.34 ± 0.52), followed by pharmacists (66.24 ± 0.58) and doctors (65.88 ± 0.44).

The results depicted higher IPEC competencies in all health professionals. On both the domains i.e., inter-professional values and inter-professional interaction, nurses outscored both pharmacists and doctors with mean scores 34.46 ± 0.26 and 32.88 ± 0.28 respectively. A detailed description is given (Table 3).

**Table 3:** Mean scores of healthcare professionals regarding IPEC competencies.

Variable	Healthcare professionals	Mean	Standard deviation	score Range	Interpretation
Inter-professional interaction	Doctors	32.38	0.25	8–40	High IPEC competencies
	Pharmacists	32.46	0.32	8–40	High IPEC competencies
	Nurses	32.88	0.28	8–40	High IPEC competencies
Inter-professional values	Doctors	33.50	0.22	8–40	High IPEC competencies
	Pharmacists	33.78	0.30	8–40	High IPEC competencies
	Nurses	34.46	0.26	8–40	High IPEC competencies
Total IPEC competencies	Doctors	65.88	0.44	16–80	High IPEC competencies
	Pharmacists	66.24	0.58	16–80	High IPEC competencies
	Nurses	67.34	0.52	16–80	High IPEC competencies

n = 500 healthcare professionals (200 doctors, 100 pharmacists, and 200 nurses).

IPEC = inter-professional education collaborative.

### 3.4. Comparison of IPEC competencies of healthcare professionals in relation to different demographics

No statistically significant difference (*P *> .05) was found in both domains of IPEC Competencies based on gender, age, hospitallocation, years of experience, and ward of the hospital. Statistically significant difference was found in the scores of the domain ‘inter-professional interaction’ based on qualification (*P *≤ .05) in which health professionals with doctorate degrees scored higher than graduates or post-graduates. However, no significant difference was found in the domain of inter-professional values based on qualification (*P *> .05). The results also revealed that nurses scored significantly higher on the domain of inter-professional values followed by pharmacists and then doctors (*P *≤ .05). However, no significant difference was found in the scores of inter-professional interaction domain based on profession (*P *> .05). A detailed description is given (Table 4).

**Table 4:** Comparison of IPEC competencies of healthcare professionals in relation to different demographics.

Variable	Composite Score	Inter-professional Interaction		Inter-professional Values	
	N	Mean rank	*P*-value	Mean rank	*P*-value
Gender	Male = 139	239.07	.271*	243.14	.469*
	Female = 361	254.90	–	253.33	–
Age in years	21–25 = 153	234.42	.529**	246.35	.284**
	26–30 = 201	256.82	–	254.12	–
	31–35 = 77	256.87	–	251.55	–
	36–40 = 43	240.72	–	217.64	–
	41–45 = 13	282.35	–	259.35	–
	46–50 = 5	302.30	–	355.80	–
	Above 50 = 8	306.38	–	325.38	–
Qualification	Graduate = 378	241.21	**.025** **	249.63	.597**
	Masters/MPhil/FCPS = 119	277.74	–	251.13	–
	Doctorate = 3	341.33	–	334.83	–
Hospital location	Lahore = 375	249.19	.644**	253.24	.473**
	Islamabad = 78	258.82	–	250.00	–
	Rawalpindi = 25	224.28	–	206.18	–
	Taxila = 22	273.20	–	255.91	–
Profession	Doctor = 200	237.06	.103**	226.10	**.001** **
	Pharmacist = 100	244.50	–	241.80	–
	Nurse = 200	266.95	–	279.26	–
Years of experience	1 to 5 years = 305	241.66	.195**	241.88	.086**
	6–10 years = 114	253.43	–	262.32	–
	11–15 years = 51	283.47	–	274.14	–
	16–20 years = 17	246.44	–	202.76	–
	More than 20 years = 13	308.15	–	318.88	–
Department	Medical = 146	245.96	.645**	240.51	.103**
	Surgical = 122	262.72	–	275.00	–
	Gynecology = 98	257.91	–	263.86	–
	Pharmacy = 82	236.36	–	236.94	–
	Orthopedic = 3	109.33	–	82.50	–
	Oncology = 22	261.66	–	240.25	–
	Psychiatry = 7	241.57	–	209.86	–
	Cardiology = 20	242.85	–	214.80	–

Bold values indicate significance difference.

IPEC = inter-professional education collaborative.

* Mann–Whitney test (*P* ≤.05).

** Kruskal–Wallis test (*P* ≤.05).

These findings suggest that while IPEC competencies appear broadly consistent across most demographic characteristics, professional role and educational level may still play a role in shaping specific aspects of IPC, particularly values-based competencies. Overall, the results highlight relatively uniform perceptions of inter-professional competencies among healthcare professionals, with limited but notable variations across profession and qualification.

## 4. Discussion

Inter-professional competencies in healthcare can be defined as “Integrated enactment of knowledge, skills, values, and attitudes that define working together across the professions, with other healthcare workers, and with patients, along with families and communities, as appropriate to improve health outcomes in specific care contexts.” These competencies are vital for safe, patient-centered, and quality delivery of healthcare services.^[2]^ The results of the current study reported that IPEC competencies significantly differ based on the disciplines of health professionals as nurses outscored both pharmacists and doctors. This is in line with the study done in Indonesia where emergency nurses had significantly more positive attitudes towards collaboration than emergency physicians.^[9]^

The IPEC competencies of doctors were found to be the lowest. When compared domain-wise, the scores were found to be statistically significant different on one of the 2 domains i.e., inter-professional values. These findings were supported by the findings of a study conducted in Germany in which nurses acquired collaborative competencies both during their undergraduate studies and training while doctors gained most of the competencies during their practice and also stated a substantial lack of acquisition of such competencies during their education.^[8]^ The gap regarding the acquisition of inter-professional values needs to be addressed in both the educational curriculum and field training, as mutual respect, shared values, and ethics serve as a foundation for achieving interdisciplinary collaboration.^[13]^

The above results highlighting the prominent differences in IPEC competencies of health professionals based on profession indicate the significance of evaluating respondents’ attitudes while planning activities related to IPE. This is because those professionals with favorable attitudes will be more likely to adopt or modify collaborative competencies as attitude determines behavior and collaborative practice.^[3]^ In the domain of inter-professional values, a large majority advocated developing a trusting relationship with other team members. This is evident from previous other studies which reported effective communication being promoted by launching trust, respect, and overcoming psychological barriers, organizing regular team-building activities and workshops to establish trust and mutual respect among healthcare professionals^[11]^ and to develop trust and respect among team members of different disciplines for IPC to be successful.^[5]^

## 5. Study limitations

As it was a survey-based study and used self-report measures to collect the data from the respondents, one of the possible limitations of this study could be some response bias which may have led to over or under-reporting of the competencies of health professionals. Moreover, difficulties in collecting the data were experienced due to busy schedule of healthcare professionals, most likely due to increased workload. There are a smaller number of hospital pharmacists generally appointed in the healthcare facilities in Pakistan, this might be a contributory factor in acquiring half of the desired sample calculated for pharmacists.

## 6. Conclusion

The present study found that nurses demonstrated higher IPEC competency scores compared to pharmacists and physicians, both overall and particularly in the domain of inter-professional values. These findings highlight existing variations in collaborative competencies across professional groups and underline the need for targeted efforts to strengthen inter-professional practice in healthcare settings. While integrating IPE at the undergraduate level is an important starting point for developing foundational competencies, it is not sufficient on its own to address the observed disparities. Improving IPC requires a continuous and structured approach that extends beyond initial training into clinical practice. Sustained development of these competencies should be supported through in-service training, workplace-based learning, and ongoing inter-professional continuing professional development programs. Embedding collaborative practices within daily clinical workflows is essential to ensure that theoretical knowledge is effectively translated into practice. In addition, institutional and organizational support is crucial in maintaining and strengthening inter-professional values. Healthcare institutions should actively promote a culture of mutual respect, shared decision-making, and open communication among all healthcare professionals. Practical strategies such as regular inter-professional workshops, simulation-based training, and team-based clinical discussions can further reinforce collaboration in real-world settings.

## Acknowledgments

The authors of this study extend their appreciation to the Ongoing Research Funding Program (ORF-2026-81), King Saud University, Riyadh 11451, Saudi Arabia.

## Author contributions

**Conceptualization:** Seemab Ashraf, Azhar Hussain, Madeeha Malik, Ayisha Hashmi, Shazia Jamshed, Long Chiau Ming.

**Data curation:** Seemab Ashraf, Azhar Hussain, Madeeha Malik, Ayisha Hashmi, Long Chiau Ming.

**Formal analysis:** Azhar Hussain, Madeeha Malik, Ayisha Hashmi, Muhammad Mamoon Iqbal, Ayesha Iqbal, Wajid Syed, Mahmood Basil A Alrawi.

**Funding acquisition:** Mohamed N Al-Arifi, Wajid Syed, Mahmood Basil A Alrawi.

**Investigation:** Seemab Ashraf, Suresh Shanmugham.

**Methodology:** Seemab Ashraf, Azhar Hussain, Ayisha Hashmi, Suresh Shanmugham, Long Chiau Ming, Ayesha Iqbal, Mahmood Basil A Alrawi.

**Project administration:** Seemab Ashraf, Azhar Hussain, Ayman M. Al-Qaaneh, Ayisha Hashmi, Muhammad Mamoon Iqbal.

**Resources:** Seemab Ashraf, Ayman M. Al-Qaaneh, Suresh Shanmugham, Shazia Jamshed, Long Chiau Ming, Muhammad Mamoon Iqbal, Ayesha Iqbal, Wajid Syed.

**Software:** Madeeha Malik, Ayman M. Al-Qaaneh, Suresh Shanmugham, Shazia Jamshed, Long Chiau Ming, Muhammad Mamoon Iqbal, Ayesha Iqbal, Mohamed N Al-Arifi, Wajid Syed.

**Supervision:** Azhar Hussain, Madeeha Malik, Suresh Shanmugham, Shazia Jamshed, Muhammad Mamoon Iqbal, Mohamed N Al-Arifi, Mahmood Basil A Alrawi.

**Validation:** Azhar Hussain, Ayman M. Al-Qaaneh, Shazia Jamshed, Long Chiau Ming, Muhammad Mamoon Iqbal, Ayesha Iqbal, Mohamed N Al-Arifi.

**Visualization:** Ayman M. Al-Qaaneh, Shazia Jamshed, Muhammad Mamoon Iqbal, Mohamed N Al-Arifi, Mahmood Basil A Alrawi.

**Writing – original draft:** Azhar Hussain, Shazia Jamshed, Wajid Syed.

**Writing – review & editing:** Seemab Ashraf, Azhar Hussain, Madeeha Malik, Ayman M. Al-Qaaneh, Ayisha Hashmi, Suresh Shanmugham, Shazia Jamshed, Long Chiau Ming, Muhammad Mamoon Iqbal, Ayesha Iqbal, Mohamed N Al-Arifi, Wajid Syed, Mahmood Basil A Alrawi.
